# Social-Emotional Learning Competencies and Problematic Internet Use among Chinese Adolescents: A Structural Equation Modeling Analysis

**DOI:** 10.3390/ijerph18063091

**Published:** 2021-03-17

**Authors:** Chun Chen, Chunyan Yang, Qian Nie

**Affiliations:** 1 Department of Counseling, Clinical, and School Psychology, University of California Santa Barbara, Santa Barbara, CA 93106, USA; chunchen@ucsb.edu; 2 Graduate School of Education, University of California Berkeley, Berkeley, CA 94720, USA; 3 Faculty of Psychology, Southwest University, Chongqing 400715, China; nieqian.edu@gmail.com

**Keywords:** problematic Internet use (PIU), social emotional learning (SEL) competencies, structural equation modeling, Chinese adolescents

## Abstract

To advance the understanding about social-cognitive factors related to Chinese adolescents’ experience with problematic Internet use (PIU), we examined the associations between social-emotional learning (SEL) competencies (i.e., responsible decision-making, social awareness, self-management, self-awareness, and social relationship) and problematic Internet use (PIU) among 1141 11th grade high school students from Southwest China. Through comparing the latent means of PIU across students with different demographic background (i.e., gender, social-economic status, left-behind status), the study found that male students endorsed higher levels of overall PIU and more problematic time management with Internet use than female students. No latent PIU mean differences were observed across family income and students’ left-behind status. Using structural equation modeling (SEM) while controlling for demographic factors, overall SEL competencies were found to have a significantly negative association with PIU. Meanwhile, all five SEL domains were also negatively associated with students’ PIU. The findings imply the importance of fostering SEL competencies in preventing PIU among Chinese adolescents. The study provides important practical implications for informing school-based SEL competencies programs for PIU prevention among Chinese youths.

Internet is ubiquitous among youth’s daily life in China. With the Internet becoming an integral tool for information and entertainment, concerns about adolescents’ problematic Internet use (PIU) emerge overwhelmingly. PIU has found to be detrimental to Chinese students’ wellbeing (e.g., poor academic performance, life satisfaction) [1,2]. Although there is a relatively high volume of research in China focusing on PIU’s detrimental consequences on adolescents’ development, little is known about the etiological factors contributing to the high prevalence rates and why adolescents differ in their PIU [2,3,4]. Previous research has recognized some social-cognitive factors (e.g., self-management; social awareness) as precursors of PIU [5,6]. Many of these social-cognitive factors were considered as components under a broader construct of social-emotional learning (SEL) competencies. However, no study has examined how SEL competencies, as a hierarchical and integrated structure, interacts with PIU among Chinese adolescents. In the present study, we aimed to address some of the research gaps by examining the associations between five core domains of SEL competencies and PIU. Findings from the present study provided vital implications to inform the development of effective SEL programs with a focus on preventing PIU among Chinese adolescents.

## 1. Social-Emotional Learning (SEL) Competencies and PIU

Given the adverse youth outcomes associated with PIU, researchers have worked on identifying risk and protective factors associated with PIU to inform PIU intervention and prevention. Individuals’ cognitive attributes have been widely studied as predictors of PIU [7]. Among the significant predictors of PIU at the individual level, many of the cognitive predictors of PIU, including self-control, impulsivity, and social skills, fell into the definition of social-emotional learning (SEL) competencies.

### 1.1. SEL Competencies

In the past decade, a number of frameworks conceptualizing SEL competencies have emerged from literature [8,9]. For example, according to the Collaborative for Academic, Social, and Emotional Learning (CASEL), social-emotional learning (SEL) is defined as “the process through which children and adults understand and manage emotions, set and achieve positive goals, feel and show empathy for others, establish and maintain positive relationships, and make responsible decisions” [10] (p. 1). SEL competencies refer to skills that enable youths to develop their SEL. To facilitate research design and program development, CASEL proposed five overarching domains of SEL [10]. The five interrelated core sets of cognitive, affective, and behavioral competencies include responsible decision making, relationship skills, self-management, social awareness, and self-awareness [11]. SEL is found to predict a range of positive school, behavioral and emotional outcomes, such as youth’s positive school engagement, bullying victimization and school violence reduction, decrease in drug use, reducing conduct problem, and less emotional disturbance [12,13,14,15,16]. SEL competencies can be taught and reinforced through evidence-based SEL programs when it takes place in a supportive school environment [17,18]. Meta-analysis research on studies evaluating evidence-based SEL programs has shown that universal school-based strategies to promote students’ SEL are a promising approach to enhance students’ success in school and life [9,14]. Among all the positivity of bolstering students’ SEL competencies, the effectiveness of SEL-focused programming on intervention and prevention of addictive behaviors stands out. From a social-cognitive perspective, PIU has been hypothesized to imply a self-regulation deficit, which suggested that PIU has similar mechanisms as addictive behaviors [19]. Thus, it is promising for researchers to understand the effect of SEL competencies on PIU, so as to help design and build evidence-based SEL programming at school to prevent adolescents’ PIU behaviors.

### 1.2. SEL Competencies in China

Even though SEL is an internationally recognized concept for youth development, because of the academic competitiveness in Chinese schools, individual socioemotional wellbeing has traditionally been neglected over academics in China [20]. SEL programs were found to be effective in various countries, such as the Second Step in the U.S. [21]. However, SEL programs have only been implemented in few major cities in China [22]. Meanwhile, this neglect of SEL in research resulted in a lack of direct evidence about the benefit of SEL competencies on youth development to convince the stakeholders to implement SEL programs in China for mitigating adolescents’ behavioral concerns [22]. Despite the fact that Chinese students’ socioemotional wellbeing has been neglected, previous research has indicated that Chinese adolescents’ social and behavioral problems, emotional disturbances, and academic achievement are highly interrelated during development [20,21]. These findings highlighted the importance of SEL competencies in youth development in China. Yet, there is still a lack of understanding of how SEL manifested in the context of Chinese youth. Meanwhile, given that previous research has found subfactors under SEL competencies to be correlated with PIU among Chinese youths, the present study aimed to understand how SEL competencies might be associated to PIU in Chinese adolescents [23].

### 1.3. SEL Subfactor: Responsible Decision Making

Responsible decision making is defined as the ability to make choices about one’s behavior and social interactions that align with ethical standards and social norms [11]. It includes making good decisions, deciding from right from wrong, and reflecting on one’s decisions. A few empirical studies have supported the linkage between responsible-decision making and PIU. However, the findings were inconsistent. For example, one study showed that excessive Internet gamers demonstrated lower decision-making ability in the Game of Dice Task than control groups in Germany college students [24]. Meanwhile, Chiou and Wan conducted a longitudinal study and found that, among adolescents from Taiwan, an increasing sense of responsibility for the aversive consequences of online gaming predicted a more considerable attitude change about online gaming overtime [25]. In contrast, another study showed that Taiwan college students with maladaptive Internet use had better decision-making skills than the control group on the Iowa gambling task [26]. The Iowa gambling task was designed to test for the ability to make decisions based on implicit emotional learning. Unlike gambling, the adverse outcomes of heavy Internet use did not occur immediately, which results in a delayed implicit emotional learning. Therefore, it was likely that PIU was not related to the part of responsible decision-making that was based on implicit emotional learning. However, they also argued that the better outcomes in the Iowa gambling task did not necessarily predict better outcome in general responsible decision-making skills in the real world. There was still unclarity about the contrasting findings. Given the limited and inconsistent findings reviewed above, more research is needed to examine the association between responsible decision making and PIU.

### 1.4. SEL Subfactor: Social Awareness

Social awareness is defined as the ability of empathy and being able to take the perspective of others [11]. It includes perspective-taking, empathy, respect for others, and appreciating diversity. Although research examining the association between social awareness and PIU was limited, one important aspect of social awareness, empathy, is a strong predictor of PIU in the limited body of existing research [27]. For example, two studies conducted on college student samples in China demonstrate a consistent relationship between empathy and PIU [27,28]. Both studies found that lower empathy was associated with higher degree of PIU. This direction of the association between empathy and PIU was also confirmed among urban left-behind children in Chongqing, China, suggesting a relationship between Chinese adolescents’ social-awareness and their PIU [29]. Those existing studies have mainly focused on empathy; none of the previous studies have examined the broader concept of social awareness in relation with PIU among Chinese adolescents.

### 1.5. SEL Subfactor: Relationship Skills

Relationship skills are defined as the ability to establish and maintain positive relationships with others [11]. It is comprised of effective communication, social relationship, and social respect. Research on relationship skills and PIU have shown mixed findings. Social skills were not recognized as a significant predictor of PIU in three longitudinal studies among grades 3–8 students in Taiwan and first-year college students in Zhejiang Province, China [30,31]. However, Wang and colleagues found that positive relationships with peers at school were a preventative factor for Chinese adolescent PIU, which suggests the positive relationship skills were associated with reduced risk of PIU [3]. Another study also found a lack of social support and loneliness are important factors to predict PIU among Chinese adolescents [32]. Meanwhile, according to the cognitive-behavioral model of PIU, low social skills, as one aspect of psychosocial problems, predisposed PIU [33]. Given the previous inconsistent findings, studies further examining the association between relationship skills and PIU among Chinese students are warranted.

### 1.6. SEL Subfactor: Self-Management

Self-management is defined as the ability to manage and regulate one’s emotions, thoughts, and behaviors [11]. It includes impulse control, behavior and emotion control, anger control, goal setting, and self-discipline. A large volume of research on identifying predictors of PIU has focused on self-management [34]. Ample research has shown that increased self-control has been associated with decreased PIU severity in the Asian population. For example, self-management was associated with decreased PIU in Chinese adolescents, and emotional regulation also had a negative association with PIU among Korean high school students [5,35,36]. The strong negative relationship between self-management and PIU has also been validated in longitudinal studies. Research has shown that a higher level of self-control was found to protect individuals from having PIU, whereas higher impulsivity predicts PIU in a four-year longitudinal study among Korean adolescents [37]. Another two-year longitudinal research of Singaporean youths found that emotional regulation, as well as impulse control, were positively associated with a lower level of PIU [38]. Although the linkage between self-management and PIU has been well supported in the literature, we have a minimal scientific understanding of self-management’ relative association with PIU, when other SEL competencies are concurrently examined. Meanwhile, the association between self-management and PIU has not been examined among Chinese left-behind adolescents. Given the consistent previous results, self-management is hypothesized to have a negative association with PIU among the current sample of Chinese adolescents.

### 1.7. SEL Subfactor: Self-Awareness

Self-awareness is defined as the ability to recognize one’s emotions, thoughts, strengths, and limitations [11]. It consists of emotion identification, strength and limitation recognition, self-esteem, self-reflection, self-efficacy, and self-confidence. Several studies demonstrated that a lower degree of self-esteem was related to a higher degree of PIU across the population in the world including being identified in East Asian youths [39,40,41]. For example, lower self-esteem was a risk factor of PIU among Chinese adolescents and Korean adolescents [37,42]. Meanwhile, lower perceived self-efficacy for reducing Internet use was found to predict PIU among middle and high school students in Hong Kong [43]. However, no previous study among Chinese adolescents has examined the entire construct of self-awareness concerning PIU. Thus, this study examined such association.

### 1.8. Associations between SEL and PIU

Although previous studies on PIU have identified several SEL-related factors as predictors, limited studies have examined the association between an integrated and hierarchical set of SEL competencies and PIU, and none was conducted in the Chinese adolescent population. Among the limited research on SEL-related predictors and PIU, only one study has examined the association between SEL competencies and PIU, with SEL competencies being measured as a multidimensional construct. The study was conducted on 1437 eighth and ninth students in Singapore to explore the relationship between adolescents’ perceptions of their regulation of a set of social-emotional competencies (i.e., task articulation, peer relationship, and self-regulation) and generalized PIU [44]. The significant relationship between overall SEL competencies and generalized PIU was not identified in the study. However, when factoring in students’ maladaptive thoughts that came from perceived academic expectations of parents and teachers as a mediator, the relationship between SEL competencies and generalized PIU (i.e., compulsive use and withdrawal) became significant [44]. However, one limitation is that simple linear regression analyses in the study was not able to recognize SEL competencies as a hierarchical structure, which was addressed in the current study.

In addition, the linkage between SEL competencies and PIU is elucidated by previous program evaluation studies on how SEL programs impact on treating addictive behaviors. For example, a meta-analysis study on drug use prevention program identified SEL to be a practical component when treating students’ substance use through developing their interpersonal/relationship skills, refusal skills, goal setting, assertiveness, communication, and coping [13]. Some researchers have argued that addictive behavioral characteristics are commonly shared between PIU and substance use [45]. It is possible that SEL competencies are also associated with PIU due to similar underlying mechanisms of addictive behaviors.

## 2. Demographic Factors and PIU

### 2.1. Age in PIU

Adolescents at high school age are a concerning population that is at the highest risk for PIU [46]. It was reported that internet consumption peaked among adolescents aged 14 to 17 [47]. Chinese high school students are particularly at a higher risk of developing maladaptive Internet use behaviors because of a range of stressors, particularly high academic pressure. Chinese high school students prevalently reported a high level of stress from school performance and the uncertainty regarding their future [48]. To enter Chinese universities, Chinese high school students must take the Chinese university entrance exam, known colloquially as “gaokao” [49]. Chinese high schools have three academic years in total. Therefore, the present study aimed to focus on 11th-grade students, who were in their high school junior year. Students in 11th grade have better adjusted to the school environment than students in 10th grade but were exposed to lesser academic stress than students in 12th grade. To control for confounding variables, the present study only looked into 11th-grade students as the sample.

### 2.2. Gender Difference in PIU

A large body of research has supported the gender differences in PIU across the world, with the male having a higher risk of PIU than females [50]. For example, among high school students in Guangzhou, China, male students were 50% more likely to demonstrate PIU symptoms than female students [51]. Another longitudinal study found that male students showed more PIU over time in Hong Kong high school students across three years [50]. Given the robust evidence that males are more prone to show PIU, it was hypothesized that male high school students were more likely to report higher levels of PIU than female students.

### 2.3. Influence of Family SES on PIU

Inconsistent results were found on the effect of family SES on youths’ PIU. Higher family income was indicated as a risk factor of PIU in a systematic review, among high school students from eight cities in mainland China and middle school students in Hong Kong [2,52,53]. However, the family economic disadvantage was found to be a risk factor of PIU among high school students in Hong Kong and among Chinese adolescents from Jilin Province, China [42,54,55]. The direction of the influence of family income on PIU was therefore uncertain and warranted further research to confirm.

### 2.4. Effect of Left-Behind Status on PIU

With the social-economic growth, a large number of migrant workers from the less developed western region came to more economically developed eastern regions for work [56]. At the same time, many of their children had to stay at home in the rural area with their grant parents or other family relatives. Those children are called “left-behind children” [57]. In 2018, the number of children left behind by one or both of their parents due to migration in China was approximately 69 million [58,59]. Although left-behind status was found to be a consistent risk factor to child development, findings supporting the impact of children’s left-behind status on PIU have been mixed. For example, one study found that left-behind children reported a rate of 10.8%, which is higher than non-left-behind children [56]. Similarly, another study found up to 18.27% of the rate of PIU among left-behind middle school students in China. The length of time parents spent at home was found to be correlated with their PIU [60]. However, Guo and the colleagues did not find differences between the prevalence rates of Chinese children who are left behind or not (3.2% vs. 3.7%) [61]. Given the present study having around 40% of left-behind participants, it is necessary to investigate the role of left-behind status on PIU in Chinese adolescents.

## 3. Theoretical Framework

The present study was guided by the cognitive-behavioral model of PIU. According to the cognitive-behavioral model of PIU, maladaptive cognitions are considered as contributors to the development and maintenance of PIU [33]. Within the context of SEL competencies, maladaptive cognitions in a youth could be manifested as the self-perceptions of their emotional competencies being low, such low self-efficacy and self-confidence. It is argued that individuals with a negative view of themselves tend to use the Internet to achieve more positive responses from others online. For individuals having PIU, self-validation through other’s positive approval in virtual settings is less intimidating and less threatening to initiate, compared to real-life communication. Besides, cognitive distortions about the world include cognitive beliefs on their social competencies within the context of SEL competencies. The model argues that distorted cognitions are automatically enacted when individuals use the Internet, especially for social purposes. Thus, maladaptive cognitions are found to be the proximal causes of PIU. However, previous studies guided by the cognitive-behavioral model mainly focused on identifying risk factors of PIU in a deficit-oriented perspective. The present study aimed to approach the cognitive-behavioral model of PIU from a strengthen-based perspective by considering the preventative effect of psychological/cognitive strengths, hence SEL competencies, in PIU.

Therefore, guided by the cognitive-behavioral model of PIU, the present study envisioned cognitive components as proximal factors that could contribute to adolescents’ risk of PIU. Davis contended that more attention should be paid to the maladaptive cognitions as the focus of understanding PIU, as it may help develop effective interventions of PIU [33]. Thus, the present study was guided by the cognitive-behavioral model to examine individuals’ perception of SEL competencies as predictors of PIU.

## 4. Study Purpose

The present study aimed to understand PIU among Chinese adolescents and the associations between an integrated and hierarchical set of SEL competencies and PIU. We first examined PIU differences among adolescents’ sex, family income status, and left-behind background. It was hypothesized that male students and students who were left behind were likely to have a higher level of PIU. Due to the inconsistent findings of family SES and students, no hypothesis was made on the relationship between family income and PIU. With the control of the demographic factors, we then examined the association between SEL competencies and its five domains and PIU. It was hypothesized that SEL competencies have a significantly negative relationship with PIU, which means that the higher a student perceived their SEL competencies, the lower level they tended to report on PIU. Within the SEL competencies, all five SEL domains are all significantly associated with PIU.

## 5. Materials & Methods

### 5.1. Participants

Participants were comprised of 1141 students from three high schools in Chongqing municipality and Sichuan province in Southwest China. All participants were at the 11th grade (*Mean* = 15.39, *SD* = 0.66). The current data was a part of an ongoing longitudinal research project conducted by researchers at Southwest University in China. Detailed demographic characteristics of the participants are presented in Table 1. Most students, comprised of 97.9%, were identified as Han ethnicity, which is the major ethnicity in China. About 25.5% of the participants’ families received a monthly income that was lower than 3000 RMB, and 63.4% received around 3000 to 10,000 RMB per month, whereas 10% received more than 10,000 RMB per month. According to the national bureau statistical report in 2019, it was documented that the average annual wage of persons employed in urban non-private units in west China, including the Southwest region where the study was conducted, was 81,954 RMB (i.e., 6829 RMB for monthly salary) [62]. Therefore, the current sample was likely to be relatively representative regarding to socio-economic status of families in Southwest China. Around 40% were identified as left-behind children, with one or both parents migrate far from home for work for more than three months.

### 5.2. Data Collection Procedure

Data were collected in February 2020. The Research Project Ethical Review Board at one of the researchers’ universities has approved all measures and procedures, with IRB number H19008. Additionally, the Research Human Subjects Department of the first author’s institutions has approved the use of the dataset, as defined in the Common Rule (45 CFR 46). The method of cluster sampling was conducted to select the participating schools from a pool of representative schools in both urban and rural areas in Southwest China. Permission was first sought from the school administrators. Questionnaires were administered either via paper forms or online form upon the choice of the schools. Paper versions were administered by researchers, research assistants, or teachers who has received training on data collection. Online questionnaires were administered from Wenjuanxing, which is a Chinese online platform equivalent to Qualtrics, with the presence of teachers in the classroom. Written consent was obtained from teachers and parents, with all student participants providing assents.

### 5.3. Measures

#### 5.3.1. Modified Chinese Version of Delaware Social and Emotional Competencies Scale—Student Version (DSECS-S)

The Modified Chinese version of Delaware Social and Emotional Competencies Scale-student version (DSECS-S) consists of 22 items on a 4-point Likert scale. The modified scale is designed on five domains: responsible decision-making (e.g., “I feel responsible for how I act.”), social awareness (e.g., “I think about how others feel.”), self-management (e.g., “I can control how I behave.”), self-awareness (e.g., “I can calm myself when upset.”), and social relationship (e.g., “I get along well with others.”). Respondents are asked to read statements to rate how applicable it is. This Chinese version was modified based on the original English version of the DSECS-S [63]. The original DSECS-S contains 16 items on only four domains, which include responsible decision-making, relationship skills, self-management, and social awareness [63]. In the present study, the current survey added the fifth domain of self-awareness based on the original survey to present the full scales of SEL. The development of the self-awareness items and the translations and back-translation of the measure were conducted by university researchers in China and one of the researchers that designed the original DSECS-S. Researchers are all fluent in English and Chinese. The DSECS-S in the current sample demonstrated a second-order five-factor model on 22 items [*χ*2(147) = 619.02, *p* < 0.001, RMSEA = 0.05, 90% CI = 0.049–0.057, CFI = 0.94]. The internal consistency coefficients are 0.84 on responsible decision-making, 0.92 on social awareness, 0.85 on self-management, 0.83 on relationship skills, and 0.88 on self-awareness. Details of the Confirmatory Factor Analysis (CFA) were presented in the Supplementary Materials.

#### 5.3.2. Modified Young’s Internet Addiction Test

The Chinese version of the IAT has been widely used and rigorously validated among Chinese youth [3,51,64]. The present study adopted the Chinese version of IAT validated in Lai et al.’s psychometric study, the current study utilized their version of the questionnaire [64]. The measure was designed to assess the problematic nature of one’s Internet use by assessing three domains of their PIU, which were (1) time management & performance (e.g., “How often do you find that you stay on-line longer than you intended?”), (2) withdrawal & social problems (e.g., “How often do you choose to spend more time on-line over going out with others?”), (3) reality substitute (e.g., “How often do you fear that life without the Internet would be boring, empty, and joyless?”). In the current study, responses on the IAT are designed to be on a six-scale option to cover a more comprehensive range of responses, from 1 (“never 未曾”) to 6 (“always 总是”). As presented in the Supplementary Materials, the IAT demonstrated a second-order three-factor model on 12 items [*χ*2(51) = 347.65, *p* < 0.001, RMSEA = 0.07, 90% CI = 0.064–0.079, CFI = 0.93]. High reliability estimates of IAT were reported in the current sample, as Cronbach’s alpha coefficients of 0.85 on time management and performance, 0.89 on withdrawal and social awareness, and 0.79 on reality substitute. The details of CFA and measurement invariance were presented in the Supplementary Materials.

#### 5.3.3. Demographic Questionnaire

Participants were asked to complete demographic information. They were asked to provide information about their sex, age, ethnicity, both father’s and mother’s education levels, family monthly income, and left-behind status.

#### 5.3.4. Analytic Plan

The analyses for the current study were conducted in a two-step series: (1) to examine whether PIU differs between students with different sex, family income, and left-behind status, and (2) to examine the relationships between SEL and its domains and PIU.

The first step was to evaluate whether PIU differs between male and female students and students from different household income levels. After achieving measurement invariance across students’ sex, family income status, and left-behind status (as shown in the Supplementary Materials), the present study compared the statistical latent mean differences of PIU across multiple groups.

**Figure 1:**
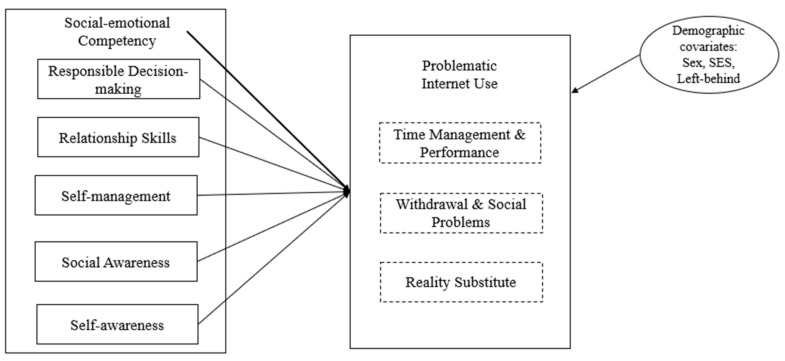
Conceptual Model: Associations Between SEL and PIU.

The second step was to evaluate the associations between SEL and its domains and PIU. The present study conducted a sequence of Structural Equation Modeling (SEM), using Mplus [65]. The proposed model is presented in Figure 1. The present study examined the associations between latent variable SEL and PIU, and the five SEL domains and PIU.

## 6. Results

### 6.1. Latent Mean Differences of PIU on Demographic Variables

Latent mean differences of PIU were tested between male and female students, across low, medium, and high family income, and between left-behind and not left-behind students. When testing latent mean differences, latent mean values of one group were set to zero, and the values of the other group were allowed to freely estimate. As observed in Table 2, the difference of latent means across students’ sex was significant (*M*_Male_ − *M*_Female_ = 0.15, d = 0.15, 95% CI [0.03, 0.31], *p* < 0.001). It indicates that the frequency of male students’ PIU was significantly higher than female students’ PIU. Among the three dimensions of PIU, male students had more frequent time management and performance concern than female students (*M*_Male_ − *M*_Female_ = 0.27, d = 0.29, 95% CI [0.18, 0.37], *p* < 0.001), but not on withdrawal and social problem and reality substitute. However, when comparing across students’ family income level and across students’ left-behind status, no differences were observed on the latent means of PIU.

### 6.2. Associations between SEL Competencies and PIU

After controlling for students’ sex, family SES, and left-behind status, a sequence of structural equation model (SEM) was conducted to examine the associations between overall SEL competencies and five SEL competencies with PIU. As shown in Table 3, the overall SEL competencies regressed on PIU with the standardized coefficient of −0.34 (.04). The SEM model was shown to be robust (*χ*2(575) = 1747.73, *p* < 0.001, RMSEA = 0.043 [90% CI: 0.041, 0.046], CFI = 0.93, TLI = 0.92, SRMR = 0.044). The association between overall SEL competencies and PIU was significantly negative. Afterwards, five separate SEM models were conducted to examine the associations between the five SEL competencies (i.e., responsible decision-making, social awareness, self-management, self-awareness, and social relationship) and PIU. All five models showed good model fits (responsible decision-making: *χ*2(175) = 710.85, *p* < 0.001, RMSEA = 0.053 [90% CI: 0.049, 0.057], CFI = 0.93, TLI = 0.91, SRMR = 0.038; social awareness: *χ*2(175) = 675.15, *p* < 0.001, RMSEA = 0.051 [90% CI: 0.047, 0.055], CFI = 0.94, TLI = 0.93, SRMR = 0.036, self-management: *χ*2(156) = 645.68, *p* < 0.001, RMSEA = 0.054 [90% CI: 0.049, 0.058], CFI = 0.93, TLI = 0.92, SRMR = 0.037, self-awareness: *χ*2(195) = 773.72, *p* < 0.001, RMSEA = 0.052 [90% CI: 0.048, 0.056], CFI = 0.92, TLI = 0.91, SRMR = 0.039, and social relationship: *χ*2(156) = 661.45, *p* < 0.001, RMSEA = 0.055 [90% CI: 0.050, 0.059], CFI = 0.93, TLI = 0.91, SRMR = 0.036). All five SEL competencies had significantly negative associations with PIU, with self-management regressing on PIU with a coefficient of −0.36, responsible decision-making with a coefficient of −0.34, social awareness with a coefficient of −0.17, self-awareness with a coefficient of −0.32, and social relationship with a coefficient of −0.28.

## 7. Discussion

The present study examined Chinese high school students’ problematic Internet use (PIU) across sex, family income level, and left-behind status, and the association between SEL competencies and PIU. First, the findings revealed that male students reported more frequent PIU than female students, but no differences were observed among the family income level and students’ left-behind status. Second, the association between overall SEL competencies and PIU was significantly negative, after controlling students’ demographic information. Also, all five SEL competencies (i.e., responsible decision-making, social awareness, self-management, self-awareness, and social relationship) and PIU were negatively associated with PIU.

### 7.1. Association between Demographic Factors and PIU

Consistent with the results of previous studies, significant gender difference was found on Chinese adolescents’ PIU experience, with male students reported higher overall IAT score than female students [51,64]. This finding was consistent with previous findings suggesting gender differences in online activities between males and females. Male students tended to use the Internet for online gaming and gambling more frequently than female students in China, which might explain the more frequent PIU reported by Chinese male students [66,67]. Another possible explanation is that in comparison to females, males tended to pass a higher threshold of Internet use to report their health and leisure life being negatively affected by the Internet than females [68]. Females might be prone to notice their PIU risks and seek help earlier than males, resulting in their lower level of PIU than males.

Under the PIU subfactors, male students in the current sample endorsed more problematic time management and performance than female students, but there was no difference in reality substitute and withdrawal and social problems between male and female students. In line with prior studies, girls were consistently found to display a stronger ability to manage and regulate their attention than boys in a meta-analysis study and among adolescents in China [69,70]. The different patterns of PIU domains across sex in this study provides insight into the manifestation of gender difference in PIU. The findings also indicated that it is important to consider adolescents’ sex in the design of PIU prevention programs.

Furthermore, family income and adolescents’ left-behind status were not associated with PIU. The insignificant association between family income level and PIU were contradicting with some previous studies in mainland China and Hong Kong, but consistent with other studies U.S., in which family income level was not a predictor of PIU [2,47,53,71]. It is possible that the magnitude of family income’s associations with PIU might vary depending on other demographic factors, such as families’ socio-cultural backgrounds. Considering the current sample is in Southwest China only, future research examining the intersections of multiple demographic factors of adolescents and their families is warranted.

Meanwhile, previous yet limited studies showed that being a left-behind Chinese youth experienced higher PIU risk, but the current finding did not find such linkage. Our literature review suggested that the risk effect of left-behind status on PIU were primarily found in studies conducted among middle school students in China [56,60]. Similarly, the only study that was conducted among Chinese high school students found no significant association between youths’ left-behind status and PIU [61]. It is possible that the contrasting findings between middle and high school students might be related to the developmental stages of participants. It is likely that as adolescents were reaching the age of adulthood, high school students were more independent and were less likely to be impacted by the absence of parental figures and parental care [72]. They become more responsible for their behaviors, which resulted in minimal effect of whether their parents are physically present on their problematic behaviors.

### 7.2. Associations between SEL Competencies and PIU

#### Overall SEL Competencies

Aligned with the hypothesis yet different from the previous study conducted among Singapore youths, a lower level of overall SEL competencies was associated with more frequent PIU in the present study. The finding is consistent with the cognitive-behavioral model of PIU, which proposes that maladaptive cognitions were sufficient proximal cause for the development of PIU [33,44]. High levels of PIU could be considered as behaviors that compensate for low levels of SEL competencies. The result provides further empirical evidence supporting that adolescents’ cognitions are strong etiological factors for PIU by highlighting that promoting students’ overall SEL competencies can be beneficial for preventing their PIU.

### 7.3. Responsible Decision-Making

Given the scarcity of literature on responsible decision-making and PIU, the present study contributed to the field by providing the initial empirical evidence supporting the association between responsible decision-making and PIU among Chinese high school students. Consistent with the previous literature on how adolescents’ decision-making was associated with their addictive behaviors (e.g., smoking), the lower level of responsible decision-making ability rendered Chinese adolescents more prone to PIU than others with a higher level of responsible decision-making. Decision-making is an inhibitory function [73]. It is possible that students with higher responsible decision-making were more likely to be conscious of their online behaviors [74]. According to the perspective of cognitive dissonance theory, when individuals feel responsible for their behavioral consequences, they would experience dissonance with what they urge to do and what they feel responsible for doing [75]. Therefore, students with higher responsible decision-making were more likely to feel responsible for the adverse outcomes resulting from their overuse of the Internet, which made them less likely to have PIU.

### 7.4. Social Awareness

In the present study, Chinese high school students’ social awareness level was significantly negatively associated with PIU. This finding is consistent with some previous findings on empathy and PIU among Chinese youth [23]. However, social awareness comprises a range of social skills, including empathy and perspective-taking. This study is among the first ones that examined the broader construct, social awareness, and PIU. It was suggested that a lower level of social skills is related to an increasing amount of Internet use, which can potentially lead to PIU [14]. Meanwhile, the dynamic relationship between social skills and PIU further results into a vicious cycle on maintaining PIU, because fewer social skills would impede individual’s ability to build meaningful social relations, which makes individuals more likely to seek compensatory online activities to cope with the loneliness or social stress [28]. Although the present study examined the broader construct of social awareness, there is a lack of study on perspective-taking and PIU. Future study is needed to investigate this research gap.

### 7.5. Self-Management

As one of the SEL competencies, self-management was shown to be a protective factor against adolescent PIU, which is aligned with the hypothesis. Consistently, the previous study has shown that Chinese students with a higher level of self-management skills tend to report a lower level of maladaptive Internet behaviors [5,70]. It is possible that adolescents with a higher level of self-control are more adept at regulating their behavioral impulses in terms of achieving long-term goals [76]. This finding supports the view that self-regulation or self-management is a promising source of adolescent resilience in the face of a heightened risk of maladaptation, which protects adolescents from developing maladaptive Internet behaviors [77]. The underlying mechanism between self-management and PIU could also be elucidated through the nature of PIU development, which is characterized as individuals’ having poorly controlled urges with their Internet behaviors, including time management and emotional regulation [78]. It is likely that individuals with low self-management tend to escape from daily activities by using the Internet [79]. Future study is suggested to examine how self-management and coping strategies impact their PIU development.

### 7.6. Self-Awareness

Consistent with the previous studies, low self-awareness was associated with Chinese high school students’ PIU. According to and the cognitive-behavioral model of PIU, low self-awareness, such as self-esteem, self-efficacy, predisposes some youths to exhibit maladaptive cognitions and behaviors that result in negative outcomes [33]. Therefore, the Internet might provide a space for their temporary relief and elicit their feelings of autonomy and competence to avoid the negativities in real life that people with low self-awareness might not want to face [80,81]. Through times, they become more habituated to use the Internet as a coping tool, which develops PIU. Meanwhile, it is possible that low self-awareness tended to lead to a sense of loss of control and addictive personality, which could cause PIU [82].

### 7.7. Relationship Skills

In line with the cognitive-behavioral model of PIU and some previous studies, relationship skills were negatively associated with PIU [32]. The current measure of relationship skills specifically asked about students’ social skills at school, instead of generally both in real life and in virtual settings. It is likely that participants with low perceived relationship skills were more likely to feel lonely in real life and use the Internet for emotional support and alleviating social negativities [83]. With the anonymity of online communication, they felt more open to making friends in virtual settings. Previous research found that multiplayer online role-playing games are significantly associated with PIU [84]. Therefore, the online space served to meet their social compensation needs [85]. However, because the current measure is only asking students’ relationships at schools, they might perceive their overall social support to be higher. Some study has reported that internet use could significantly increase general perceived social support [86]. Because of the discrepancies of social support from school and virtual settings, students who did not perceive good relationships at school would be more likely to be indulged in online relationships, which results in PIU.

## 8. Limitations and Future Research Directions

The results of the study should be taken with consideration of some limitations. First, both DSECS-S and IAT are self-report measures. Self-report measures could be influenced by social desirability. Future studies can use multi-informant measures and qualitative methods to collect more insightful and comprehensive perspectives of students’ Internet use behaviors. Second, the cross-sectional research design in the study did not allow making causality between the variables. The present study would function as a pilot study to inform the future longitudinal study, in which more investigation will be taking place in understanding the reciprocal relationship between SEL competencies and PIU. Third, the generalizability of the results to Chinese high school students is limited by the sample because all students were recruited only at one grade (i.e., 11th grade) from three high schools in two provinces in Southwest China. The data collected in the study was relatively within a low to middle level of socio-economic status (SES) compared to the entire population of Chinese families. It did not reflect a wide range of SES. Replication in the future study using a more diverse and representative sample is required for generalization.

## 9. Conclusions and Implications

Using the cognitive-behavioral framework of PIU, the study examined the relationships between SEL competencies and PIU among Chinese high school students. The findings of the present study have important implications to both the theory and practice related to PIU prevention. It is one of the first studies testing the cognitive-behavioral framework based on the empirical examination of PIU’s association with SEL competencies, as a multidimensional and hierarchical construct supported by the CASEL’s SEL model among Chinese adolescents. As for the practical implications, the study informs school-based practical implications for PIU prevention among Chinese adolescents. Firstly, the protective factors of the overall SEL competencies and all five core SEL skills were confirmed. School-wide SEL programs have been found to be effective in fostering students’ psychosocial behaviors and minimizing their risks in problematic behaviors [12,14]. However, the application of SEL programs to be implemented in China is still limited. The present study confirms that SEL competencies are a meaningful quality construct in Chinese adolescents, and it highlights the importance of promoting Chinese students’ SEL. This observation suggests that programs targeting at either overall or specific domain of SEL competencies can be useful in preventing high school students’ problematic Internet use. On the school level, SEL curriculums or activities that foster students’ SEL could be considered to implement in class. On the individual level, the findings imply that treatment aimed at improving adolescents’ social-emotional cognition, such as cognitive behavioral therapy, may also be promising for reducing the development of PIU. For example, school-based training, such as impulse-control techniques, time management, and interpersonal communication skills, have shown to be effective in preventing PIU among Chinese students [87]. Also, the significant effects of gender and other demographic factors on PIU indicate that it is important to take the demographic factors of students into consideration when designing effect PIU prevention programs in schools.

## Figures and Tables

**Table 1:** Participants’ Demographic Characteristics.

Characteristics	N	%
11th Grade	1141	100%
Sex		
*Male*	545	47.8%
*Female*	583	51.1%
Ethnicity in China		
*Han ethnicity*	1117	97.9%
*Ethnic minorities*	15	1.3%
Left-behind Status		
*Both parents working outside*	226	19.8%
*Only father working outside*	188	16.5%
*Only mother working outside*	30	2.6%
*Not left-behind*	685	60%
Family monthly income		
*Lower than ¥3000*	291	25.5%
*¥3000–10,000*	723	63.4%
*Greater than ¥10,000*	114	10%
Parents’ educational degrees		
Father’s educational degree		
*Elementary school degree and below*	195	17.1%
*Junior high school degree*	532	46.6%
*High school degree and special degree*	298	26.1%
*Undergraduate degree*	1000	8.8%
*Graduate degree and above*	4	0.4%
Mother’s educational degree		
*Primary school degree and below*	270	23.7%
*Junior middle school degree*	507	44.4%
*High school degree and special degree*	282	24.7%
*Undergraduate degree*	65	5.7%
*Graduate degree and above*	1	0.1%

**Table 2:** Latent and Observed Mean Differences of Problematic Internet Use (PIU).

Latent Mean and Differences				Observed Means	
*M*_V1._-*M*_V2_				V1	V2
	Estimates	*d*	*d*	*M (SD)*	*M (SD*)
			(95% CIs)		
**Problematic Internet Use (PIU)**					
Male (V1)−Female (V2)	0.15 **	0.15	[0.03, 0.31]	2.36 (1.07)	2.24 (0.98)
Low income (V1)−Middle income (V2)	−0.10	−0.10	[−0.26, 0.04]	2.22 (0.97)	2.32 (1.02)
Middle income (V1)−High income (V2)	0.04	0.04	[−0.21, 0.24]	2.32 (1.02)	2.27 (1.15)
Low income (V1)−High income (V2)	−0.06	−0.06	[−0.35, 0.16]	2.22 (0.97)	2.27 (1.15)
Left-behind (V1)−Not left-behind (V2)	0.05	0.05	[−0.08, 0.17]	2.32 (1.05)	2.26 (1.01)
**Time Management & Performance**					
Male (V1)−Female (V2)	0.27 **	0.29	[0.18, 0.37]	2.60 (1.18)	2.56 (1.16)
**Withdrawal & Social Problems**					
Male (V1)−Female (V2)	0.02	0.02	[−0.13, 0.13]	2.10 (1.07)	1.84 (0.87)
**Reality Substitute**					
Male (V1)−Female (V2)	0.11	0.10	[−0.04, 0.21]	2.36 (1.25)	2.26 (1.19)

** *p* < 0.001.

**Table 3:** Standardized Coefficients for the Relations Between Social-Emotional Learning (SEL) Competencies and Problematic Internet Use (PIU).

Outcome	Predictor	PIU Coefficients	
		β (SE)	B (SE)
Problematic Internet Use (PIU)	Sex (0 as male)	−0.10 (0.03) **	−0.18 (0.06) **
	Family Income	0.01 (0.03)	0.02 (0.05)
	Left-behind Status	−0.02 (0.03)	−0.02 (0.02)
	Father Education Level	−0.01 (0.04)	−0.02 (0.04)
	Mother Education Level	0.01 (0.04)	0.01 (0.04)
	SEL Competencies	−0.34 (0.04) ***	−0.81 (0.09) ***
	Responsible Decision-making	−0.34 (0.04) ***	−0.73 (0.09) ***
	Social Awareness	−0.17 (0.04) ***	−0.33 (0.07) ***
	Self-management	−0.36 (0.04) ***	−0.59 (0.06) ***
	Relationship Skills	−0.28 (0.04) ***	−0.47 (0.06) ***
	Self-awareness	−0.32 (0.04) ***	−0.62 (0.07) ***

Note. Wald χ^2^ tests were conducted to determine whether coefficients for the United States and China were significantly different. For all Wald χ^2^ tests, *df* = 1. *SE* = standard error. β = standardized coefficient. Β = unstandardized coefficient. ** *p* < 0.005, *** *p* < 0.001.

## Data Availability

Data available on request due to privacy restrictions.

## Supplementary Materials

The following are available online at https://www.mdpi.com/1660-4601/18/6/3091/s1, Confirmatory Factor Analyses and Measurement Invariances: DSECS-S and IAT.
