# Supplementary material for: Social-Emotional Learning Competencies and Problematic Internet Use among Chinese Adolescents: A Structural Equation Modeling Analysis
Source: Int J Environ Res Public Health. 2021 Mar 17;18(6):3091. doi: 10.3390/ijerph18063091 (PMC8002824; doi:10.3390/ijerph18063091)
Supplement: Supplementary file 1 [file ijerph-18-03091-s001.pdf]

Supplemental Material: Confirmatory Factor Analyses and Measurement Invariances: DSECS-S and IAT

Modified Chinese Version of Delaware Social and Emotional Competencies Scale – Student Version (DSECS-S)

The study validated the factor structures of the modified Delaware Social and Emotional Competencies Scale – Student version (DSECS-S) and the modified Young's Internet Addiction Test (IAT) in Chinese versions, in order to make sure that the key measures used for the study were psychometrically sound for the Chinese high school students of the present study.

The first step was to evaluate the construct validity of DSECS-S among Chinese high school students. Confirmatory factor analyses (CFA) was conducted to evaluate proposed models, using Mplus 7.4 [1]. Based on the theory and definition of SEL competencies, the present study hypothesized a second-order five-factor model. To compare across with other factor models, the current study also conducted CFA on the five-factor correlated model, one-factor model, and bifactor model. Fit indices were relied on to determine model fit. A good model would be considered, if the comparative fit index (CFI) was close to 0.95, Tucker-Lewis Index (TLI) is close to 0.95, the root mean square error approximation (RMSEA) was <=.06, and the standardized root-mean-square residual (SRMR) was <=.07 [2,3]. The reason why the cutoff of SRMR is 0.07, instead of 0.08, as the standard rule of thumb, is that, when the sample size is larger than 250, the cutoff of 0.07 might be better to retain reasonable power in complex models [3]. It was recommended to use SRMR, supplemented by TLI, CFI, or RMSEA [3]. If more than one models fit the criteria, the model with best-fit indices and/or most aligned with the definition of SEL competencies was selected.

Modified Young's Internet Addiction Test

The second step was to evaluate the construct validity of the modified IAT. CFA was conducted to measure to evaluate the proposed models. Informed by the definition of PIU in the present study and the previous validated model structure, a second-order three-factor model is hypothesized, presented in Supplemental Material Figure 2 [4]. Similarly, a good model would be considered, if the comparative fit index (CFI) was close to 0.95, Tucker-Lewis Index (TLI) is close to 0.95, the root mean square error approximation (RMSEA) was <=.06, and the standardized root-mean-square residual (SRMR) was <=.07 [2,3]. The present study compared and contrasted fit indices across the second-order three-factor model, bifactor model, three-factor model, and one-factor model.

Because the present study aimed to compare the latent mean differences of PIU among participants' demographic variables, in the third step, we conducted measurement invariance of the modified IAT measure among students' sex, family income levels, and left-behind status.

Results

Factor Structure and Measurement Invariance

Modified DSECS-S

CFA was run on the 22 items in the DSCES-S. Table 1 presents the fit indexes for the five-factor, second-order, one-factor and bifactor models. Both six-factor model and second-order model of DSECS-S reported adequate goodness-of-fit. After the model comparison, insignificant results in the SB χ^2 differences were obtained. Nevertheless, the second-order model was selected, as the model structure was mostly consistent with the definition of SEL competencies. Due to low factor loadings of three items (i.e., items 19, 16, and 21), the final factor structure model indicated a second-order model structure on 19 items. The higher-order factor was shown to be SEL competencies. The five lower-order factors were responsible decision-making, social awareness, self-management, relationship skills, and self-awareness.

Modified IAT

CFA was conducted on 13 items in the IAT. The second-order three-factor model, three-factor model, one-factor model, and bifactor model were tested. As shown in Table 6, both three-factor model and second-order model had the best fit indices, and no differences were observed after model comparison. Based on the definition of PIU, the second-order three-factor model was selected. One item was dropped due to its low factor loading. As shown in Table 7, 8, and 9, full scalar measurement invariance was achieved across students' family income level. However, partial metric invariances were achieved across students' sex and left-behind status, after freeing a few item loadings. Thus, as the hypothesized model structure, the final model has PIU as the second-order factor, and time management and performance, withdrawal and social problems, and reality substitute as the three lower factors. Internal consistency coefficients were calculated on the modified IAT. The alpha coefficient of time management and performance is 0.85; the alpha coefficient of withdrawal and social awareness is 0.89; and the alpha coefficient of reality substitute is 0.79.

Discussion

Psychometric Properties of DSECS-S and IAT

The present study supported that the modified Chinese version of DSECS-S showed a second-order five-factor structure, which is consistent with the model structure in the English version of the DSECS-S [5]. This replication indirectly suggested the stability of the second-order five-factor structure of the SEL concept from U.S. youth to Chinese adolescents. However, a cross-cultural comparison study is warranted to test such a hypothesis.

In addition, the modified Chinese version of IAT presented a second-order three-factor structure, similar to the model proposed in the previous studies [4]. Although several studies have been using IAT to assess the severity of Internet use problems in Chinese adolescents, there has been only one study that has established a factorial structure of IAT among Hong Kong adolescents [4,6]. Therefore, the current measure validation of IAT is among the first studies that supported the application and explored the dimensionality of IAT among mainland

Chinese adolescents. This replication from Lai et al.'s study indirectly suggested the stability of the second-order three-factor structure of IAT in adolescents. Compared to the original Young's IAT, which was initially designed as a single-dimension instrument for diagnostic purposes, the present study supported a hierarchical multidimensional construct as the best fitting model among the participants [7]. The multidimensional structure of the modified IAT in the present study implies and enriches the notion of PIU. PIU might be better represented as a constellation of correlated symptoms. For example, an individual might have greater difficulty with time management on the Internet but have a low tendency to substitute reality with the Internet. Future research and practice could lend support to the total score of IAT as a determinant of the PIU severity, as well as relying on the multidimensional constructs for in-depth information on an individual's PIU manifestation. Besides, full measurement invariance was achieved across students' family income level, whereas partial metric invariances were achieved across their sex and left-behind status. However, the previous measurement study had suggested that the same unbiased patterns of findings continued to be observed when they employed the corrected partial metric invariance model to fit their dataset[8]. Therefore, the interpretations of the latent mean differences in PIU need to be cautious in generalization.

References

1. Muthén, L. K., & Muthén, B. O. (1998–2015). *Mplus user's guide* (7th ed.). Los Angeles, CA: Authors.
2. Hu, L. T., & Bentler, P. M. (1998). Fit indices in covariance structure modeling: Sensitivity to underparameterized model misspecification. *Psychological Methods, 3*, 424–453.
3. Yu, C. Y. (2002). *Evaluating cutoff criteria of model fit indices for latent variable models with binary and continuous outcomes* (Vol. 30). Los Angeles: University of California, Los Angeles.
4. Lai, C. M., Mak, K. K., Watanabe, H., Ang, R. P., Pang, J. S., & Ho, R. C. (2013). Psychometric properties of the internet addiction test in Chinese adolescents. *Journal of Pediatric Psychology, 38*, 794–807. https://doi.org/10.1093/jpepsy/jst022
5. Author et al., 2016
6. Lam, L. T., Peng, Z. W., Mai, J. C., & Jing, J. (2009). Factors associated with Internet addiction among adolescents. *Cyberpsychology & Behavior, 12*, 551–555. https://doi.org/10.1089/cpb.2009.0036
7. Young, K. S. (1998). Internet addiction: The emergence of a new clinical disorder. *Cyberpsychology & Behavior*, *1*, 237–244. https://doi.org/10.1089/cpb.1998.1.237
8. Hsiao, Y. Y., & Lai, M. H. (2018). The impact of partial measurement invariance on testing moderation for single and multi-level data. *Frontiers in Psychology, 9*, 740. https://doi.org/10.3389/fpsyg.2018.00740

6. Lam, L. T., Peng, Z. W., Mai, J. C., & Jing, J. (2009). Factors associated with Internet addiction among adolescents. *Cyberpsychology & Behavior, 12*, 551–555. https://doi.org/10.1089/cpb.2009.0036

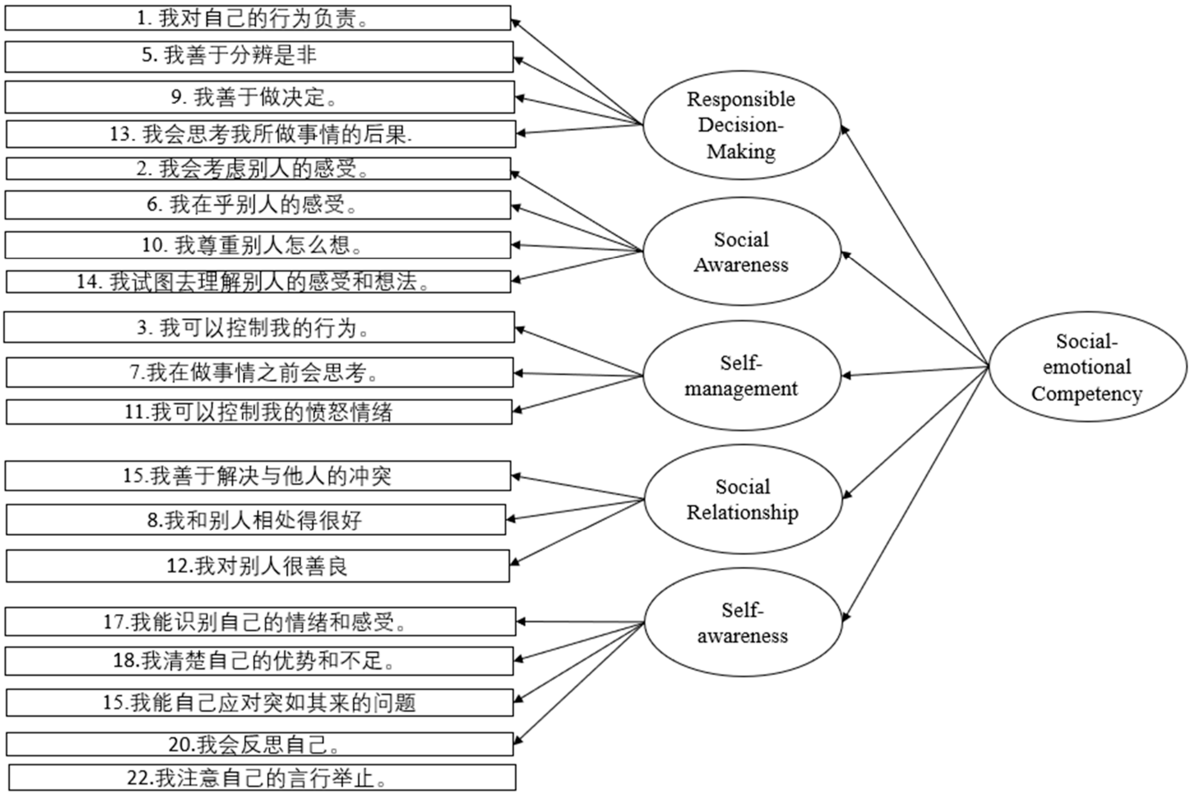

Figure S1. Second-order five-factor model.

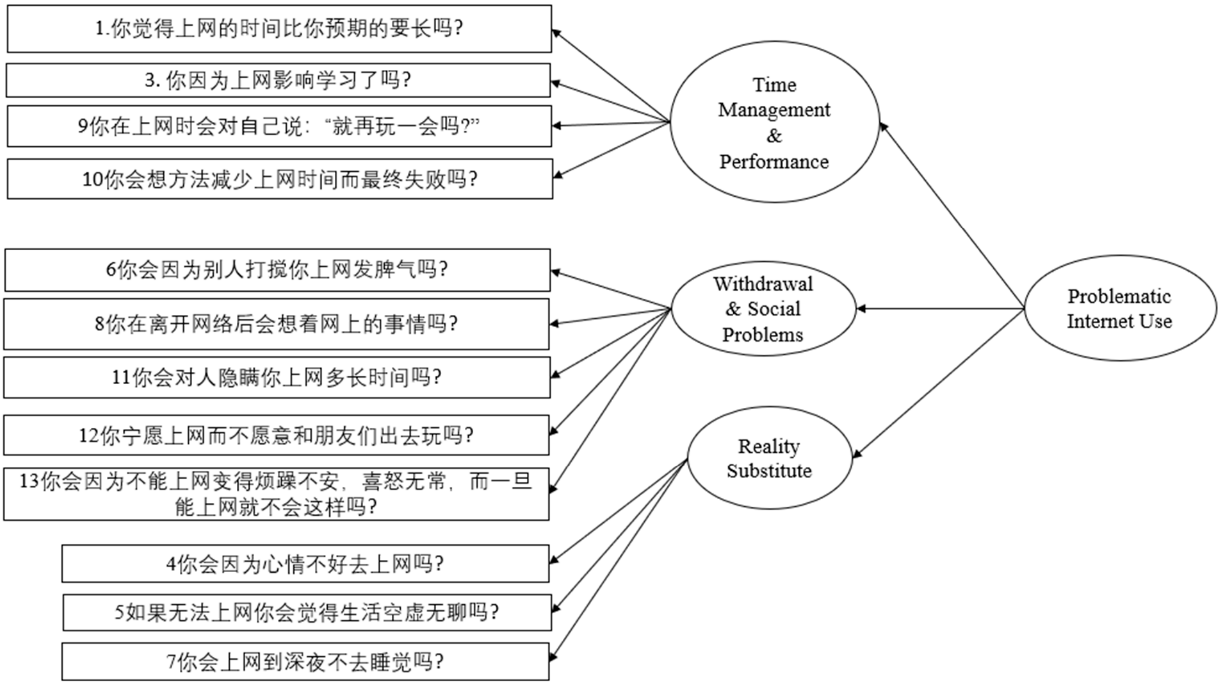

Figure S2. Second-order three-factor model.

Table S1. Goodness-of-Fit Indicators of Models for DSECS-S.

Model	χ^2	*df*	SRMR	RMSEA [90% CI]	CFI	Model Comparison	Δ $S\text{-}B\chi^2$	Δ *df*	Δ CFI
**Model 1:** Second-order model	619.02*	147	0.042	0.053 [0.049, 0.057]	0.941	2 vs. 1	19.72	5	0.002
**Model 2:** Six-factor model	599.36*	142	0.040	0.053 [0.049, 0.058]	0.943				
**Model 3:** One-factor model	1659.55*	153	0.065	0.093 [0.089, 0.097]	0.811				

Note. CFI = Comparative Fit Index; SRMR = Standardized Root Mean- Square Residual; RMSEA = Root Mean-Square Error of Approximation. Models were tested on full sample. Bifactor model was not converged. *$p < 0.001$.

Table S2. Goodness-of-Fit Indicators of Models for IAT.

Model	χ^2	*df*	SRMR	RMSEA [90% CI]	CFI	Model Comparison	Δ $S\text{-}B\chi^2$	Δ *df*	Δ CFI
**Model 1:** Second-order model	347.65*	51	0.043	0.071 [0.064, 0.079]	0.931	3 vs. 1	96.60*	3	0.029
**Model 2:** Three-factor model	347.65*	51	0.043	0.071 [0.064, 0.079]	0.931				
**Model 3:** One-factor model	472.75*	54	0.049	0.082 [0.076, 0.089]	0.902				

Note. CFI = Comparative Fit Index; SRMR = Standardized Root Mean- Square Residual; RMSEA = Root Mean-Square Error of Approximation. Models were tested on full sample. Bifactor model was not converged. *$p < 0.001$.

Table S3. Fit Statistics for IAT Measurement Invariance across Sex.

Comparison by Timepoints	χ^2	*df*	SRMR	RMSEA [90% CI]	CFI	Model Comparison	Δ $S\text{-}B\chi^2$	Δ *df*	Δ CFI
**Model 1**: configural invariance	412.18**	108	0.046	.071 [.064, 0.078]	0.930	—	—	—	—
**Model 2**: first-order factor loadings invariant	434.12**	114	0.049	.071 [.064, 0.078]	0.926	2 vs. 1	21.93*	6	0.004
**Model 3**: first- and second-order factor loadings invariant	430.60**	110	0.059	.072 [.065, 0.079]	0.926	3 vs. 2	31.7	4	0.000
**Model 4:** first- and second-order factor loadings and intercepts of measured variables invariant	439.72**	112	0.059	.072 [.065, 0.079]	0.925	4 vs. 3	10.51*	2	0.001
**Model 5:** first- and second-order factor loadings, and intercepts of measured variables and first-order factors invariant	462.34**	115	0.063	.073 [.066, 0.080]	0.920	5 vs. 4	32.62*	3	0.005

*$p < 0.05$, ** $p<0.001$.

Table S4. Fit Statistics for IAT Measurement Invariance across Family Income.

Comparison by Timepoints	χ^2	*df*	SRMR	RMSEA [90% CI]	CFI	Model Comparison	Δ $S\text{-}B\chi^2$	Δ *df*	Δ CFI
**Model 1**: configural invariance	509.10**	153	0.047	.079 [.071, 0.086]	0.925	—	—	—	—
**Model 2**: first-order factor loadings invariant	525.16**	165	0.049	.076 [.069, 0.084]	0.924	2 vs. 1	8.62	12	0.001
**Model 3**: first- and second-order factor loadings invariant	528.69**	169	0.050	.075 [.068, 0.083]	0.924	3 vs. 2	1.71	4	0.000
**Model 4:** first- and second-order factor loadings and intercepts of measured variables invariant	538.26**	173	0.051	.075 [.068, 0.082]	0.923	4 vs. 3	6.64	4	0.001
**Model 5:** first- and second-order factor loadings, and intercepts of measured variables and first-order factors invariant	564.74**	185	0.054	.074 [.067, 0.081]	0.920	5 vs. 4	21.57*	12	0.003

*$p < 0.05$, ** $p<0.001$.

Table S5. Fit Statistics for IAT Measurement Invariance across Left-behind Status.

Comparison by Timepoints	χ^2	*df*	SRMR	RMSEA [90% CI]	CFI	Model Comparison	Δ $S\text{-}B\chi^2$	Δ *df*	Δ CFI
**Model 1**: configural invariance	446.95**	108	0.047	0.075 [0.068, 0.082]	0.922	—	—	—	—
**Model 2**: first-order factor loadings invariant	466.37**	121	0.049	0.071 [0.065, 0.078]	0.921	2 vs. 1	13.41	15	0.001
**Model 3**: first- and second-order factor loadings invariant	440.32**	113	0.048	0.072 [0.065, 0.079]	0.925	3 vs. 2	16.77	10	0.004
**Model 4:** first- and second-order factor loadings and intercepts of measured variables invariant	445.10**	115	0.048	0.072 [0.065, 0.079]	0.924	4 vs. 3	2.31	2	0.001
**Model 5:** first- and second-order factor loadings, and intercepts of measured variables and first-order factors invariant	543.68**	141	0.054	0.072 [0.065, 0.078]	0.915	5 vs. 4	97.05*	26	0.009

*$p < 0.05$, ** $p < 0.001$.
